# Supplementary material for: A non-canonical vitamin K cycle is a potent ferroptosis suppressor
Source: Nature. 2022 Aug 3;608(7924):778–83. doi: 10.1038/s41586-022-05022-3 (PMC9402432; doi:10.1038/s41586-022-05022-3)
Supplement: Supplementary file 1 — Supplementary Tables 1–3 and Supplementary Figs. 1–3. [file 41586_2022_5022_MOESM1_ESM.pdf]

nature portfolio

https://doi.org/10.1038/s41586-022-05022-3

Supplementary information

A non-canonical vitamin K cycle is a potent ferroptosis suppressor

In the format provided by the authors and unedited

nature portfolio

https://doi.org/10.1038/s41586-022-05022-3

Supplementary information

A non-canonical vitamin K cycle is a potent ferroptosis suppressor

In the format provided by the authors and unedited

A non-canonical vitamin K cycle is a potent ferroptosis suppressor

Eikan Mishima*, Junya Ito, Zijun Wu, Toshitaka Nakamura, Adam Wahida, Sebastian Doll, Wulf Tonnus, Palina Nepachalovich, Elke Eggenhofer, Maceler Aldrovandi, Bernhard Henkelmann, Ken-ichi Yamada, Jonas Wanninger, Omkar Zilka, Emiko Sato, Regina Feederle, Daniela Hass, Adriano Maida, André Santos Dias Mourão, Andreas Linkermann, Edward K. Geissler, Kiyotaka Nakagawa, Takaaki Abe, Maria Fedorova, Bettina Proneth, Derek A. Pratt & Marcus Conrad*

***Correspondence**: eikan@med.tohoku.ac.jp (E.M.) or marcus.conrad@helmholtz-muenchen.de (M.C.)

Table of Contents:

Supplementary Table 1. Sequences of sgRNA guides used to generate the knockout cell lines.
Supplementary Table 2. Analytical condition of FIA-MS analysis for detection of MK4-H2.
Supplementary Table 3. Summary of the potency of vitamin K for ferroptosis inhibition.

Supplementary Fig 1. Uncropped gel images.
Supplementary Fig 2. NMR spectrums of dimethylmenadione, VK-coumarin and CoQ-coumarin.
Supplementary Fig 3. An example of the gating strategy in Bodipy C-11 assay

Supplementary Video 1. Live imaging of HT1080 cells treated with RSL3 (0 - 8 h after addition of RSL3).
Supplementary Video 2. Live imaging of HT1080 cells cotreated with RSL3 + MK4 (0 - 8 h after addition of RSL3).

Supplementary Table 1. Sequences of sgRNA guides used to generate the knockout cell lines. Nucleotides in italics show the overhangs introduced into oligos that are necessary for cloning in the *BsmB*I restriction sites of lentiCRISPR v2 (LCv2) or *Bbs*I site of pKLVU6gRNA(BbsI) vectors.

Parental cell	Cell name	Backbone vector	Target gene	sgRNA (5'-)	Cas 9
A375	A375 GPX4^{KO}	LCv2_blast	*hGPX4*	*cacc*GCGTGTGCATCGTCACCAACG	Transient
		LCv2_puro		*cacc*GCACGCCCGATACGCTGAGTG	
786-O	786-O GPX4^{KO}	LCv2_blast	*hGPX4*	*cacc*GCGTGTGCATCGTCACCAACG	Stable
		LCv2_puro		*cacc*GCACGCCCGATACGCTGAGTG	
B16F10	B16F10 GPX4^{KO}	LCv2_blast	*mGpx4*	*cacc*GCATGCCCGATATGCTGAGTG	Transient
		LCv2_puro		*cacc*GCGTGTGCATCGTCACCAACG	
B16F10 GPX4^{KO}	B16F10 GPX4^{KO} /FSP1^{KO}	LCv2_blast	*mFsp1*	*cacc*GCCGTGCACGTGGTGATCGT	Transient
		LCv2_puro		*cacc*GCCCACGATCACCACGTGCA	
HepG2	HepG2 FSP1^{KO}	LCv2_puro	*hFSP1*	*cacc*GGGAATCGGGAGCTCTGCACG	Stable
		LCv2_puro		*cacc*GGGTGCAGAGAATCACCAGGT	
786-O _pCW-Cas9-blast	786-O FSP1^{KO}	pKLV-U6gRNA	*hFSP1*	*cacc*GGTCTTTTTGGCGAACCCTG*gt*	Dox-inducible
HT1080 _pCW-Cas9-blast	HT1080 FSP1^{KO}	pKLV-U6gRNA	*hFSP1*	*cacc*GGTCTTTTTGGCGAACCCTG*gt*	Dox-inducible

Supplementary Table 2. Analytical parameters of FIA-MS analysis for the detection of MK4-H_2.

Source	Electrospray ionization (ESI)
Ion polarity	Positive
Scan type	Enhanced MS
Scan rate (Da/s)	10000
Mass range (m/z)	200–600
Declustering potential (V)	60.00
Entrance potential (V)	10.00
Collision energy (V)	10.00
Collision cell exit potential (V)	0.00
Curtain gas (psi)	30
Ion spray voltage (V)	5500
Temperature (oC)	450
Ion source gas 1 (psi)	60
Ion source gas 2 (psi)	60
Flow rate (mL/min)	0.4
Mobile phase	Methanol

Supplementary Table 3. Summary of the potency of vitamin K for ferroptosis inhibition

		IC50 (μM) for inhibiting ferroptosis			
Cells	Induction of ferroptosis	PK	MK4	Menad	Dimethyl-menad
Pfa1	*Gpx4* deletion by TAM	0.18	0.074	1.4	No inhibition
A375 $GPX4^{KO}$	Lip1 withdrawal	0.061	0.016	0.47	
786O $GPX4^{KO}$	Lip1 withdrawal	0.49	0.078	1.3	
786O wt	RSL3	0.49	0.39	1.1	
786O $FSP1^{KO}$	RSL3	6.6	5.5	3.7	
786O $FSP1^{KO}$ + $hFSP1^{OE}$	RSL3	0.50	0.56	1.5	

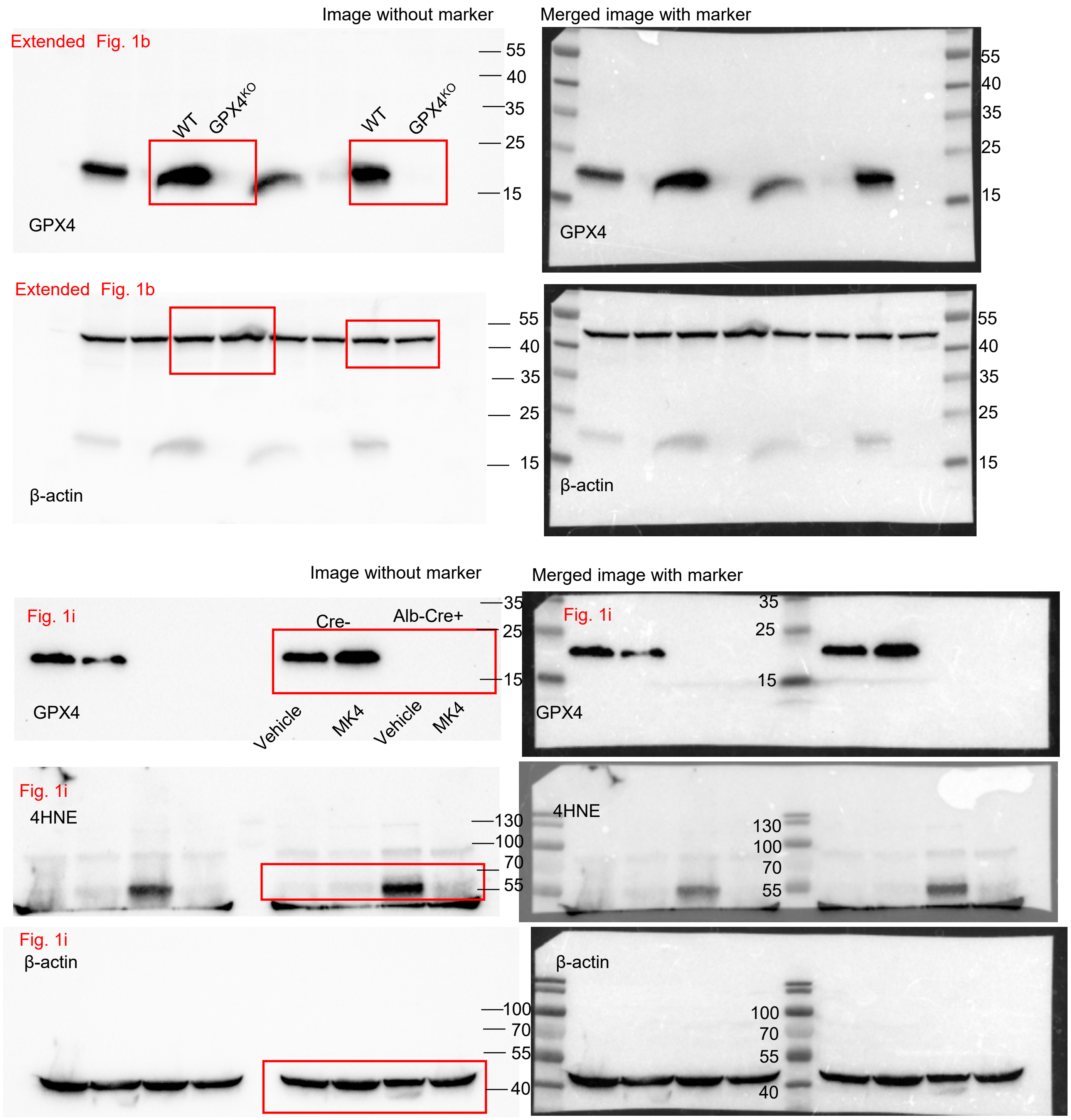

Supplementary fig. 1. Uncropped gel images.

Raw images of immunoblotting and the images merged with a molecular marker image were shown. Images with the VCP antibody were shown as the raw images with the molecular mass information of 90 kD attached at the time of image acquisition. In the images with the VCP antibody, only a clear single band at the predicted molecular mass size (97 kD) can be detected across the whole membrane as shown in the raw image of extended fig. 10b.

Fig. 4a

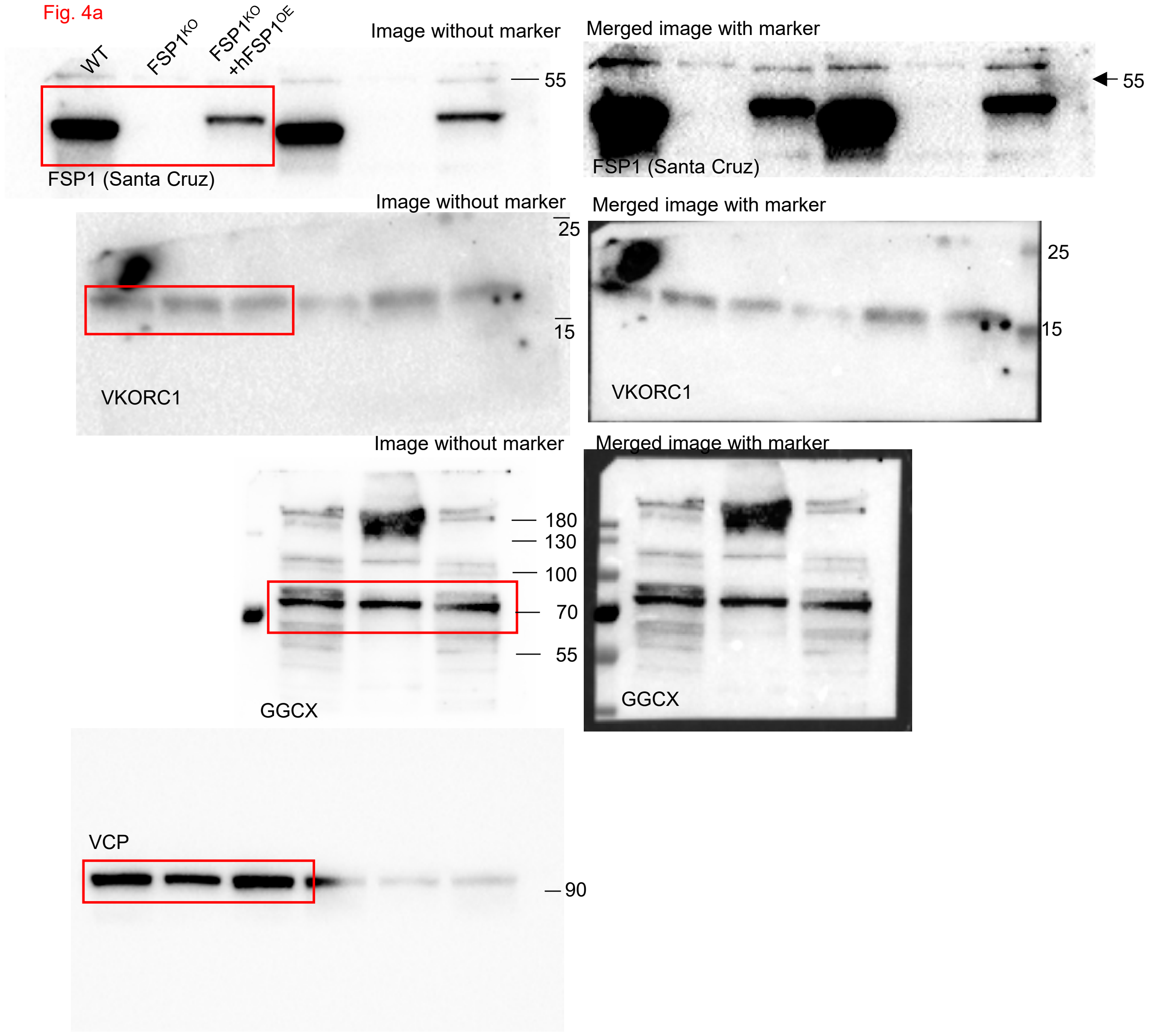

Supplementary fig. 1 (continued)

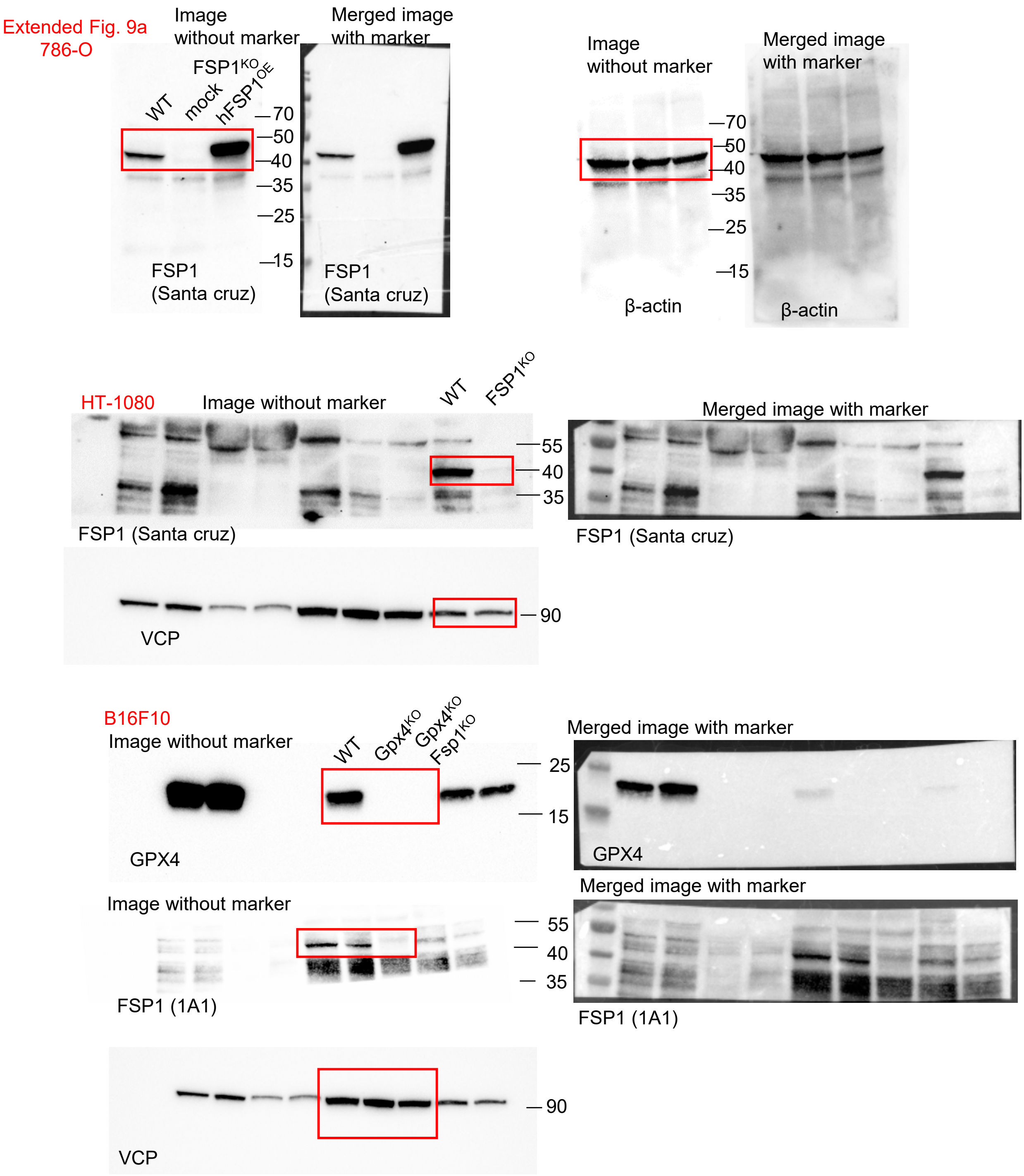

Supplementary fig. 1 (continued)

Extended Fig. 9f

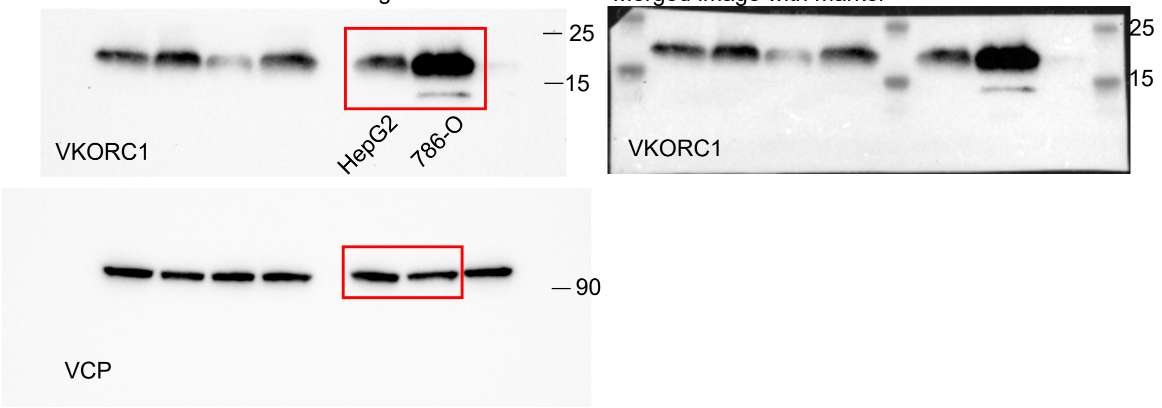

Extended Fig. 9d

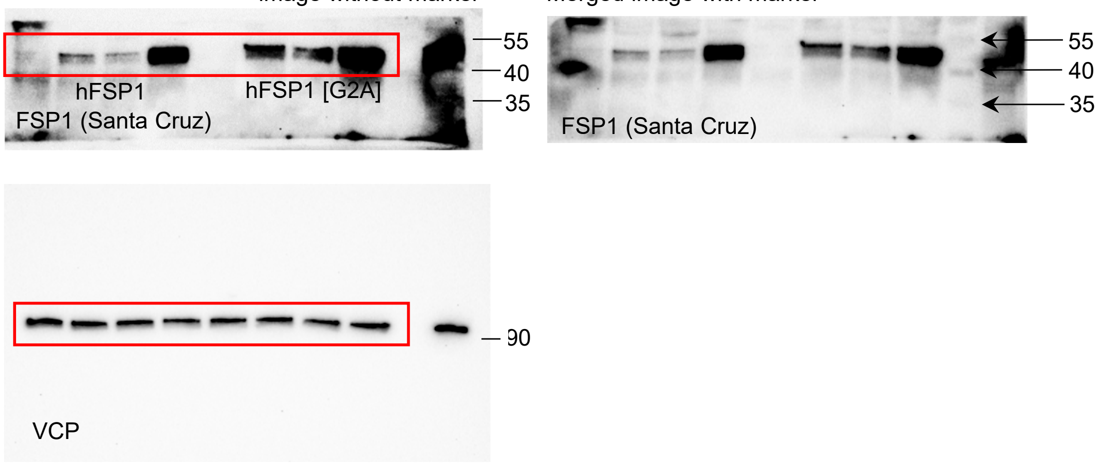

Extended Fig. 10a

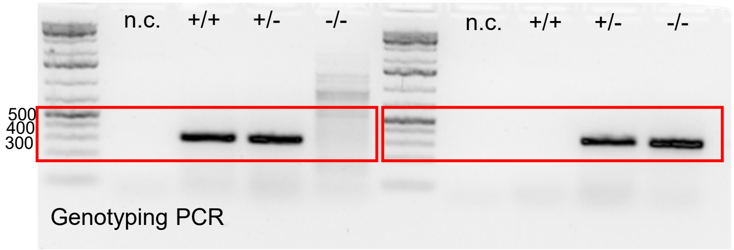

Extended Fig. 10b

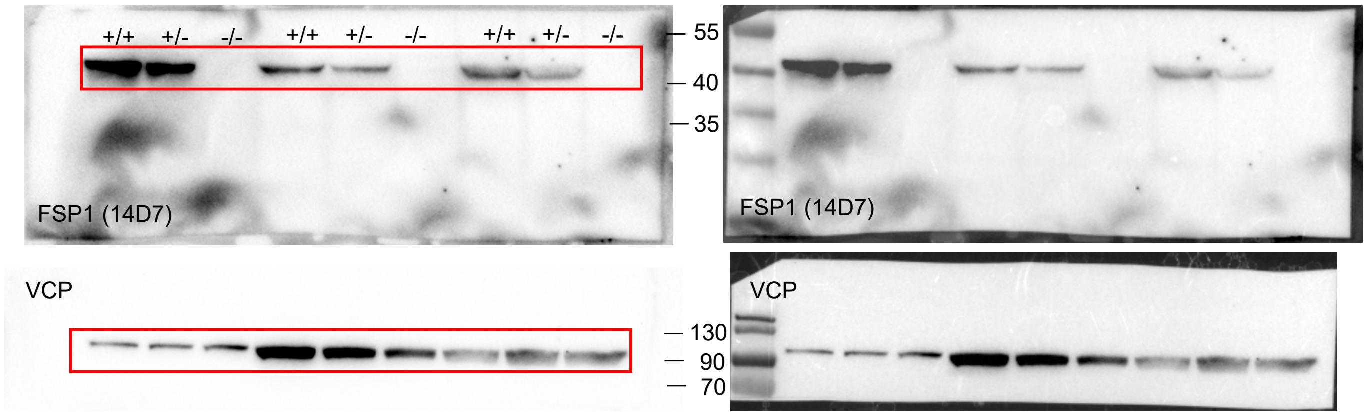

Supplementary fig. 1 (continued)

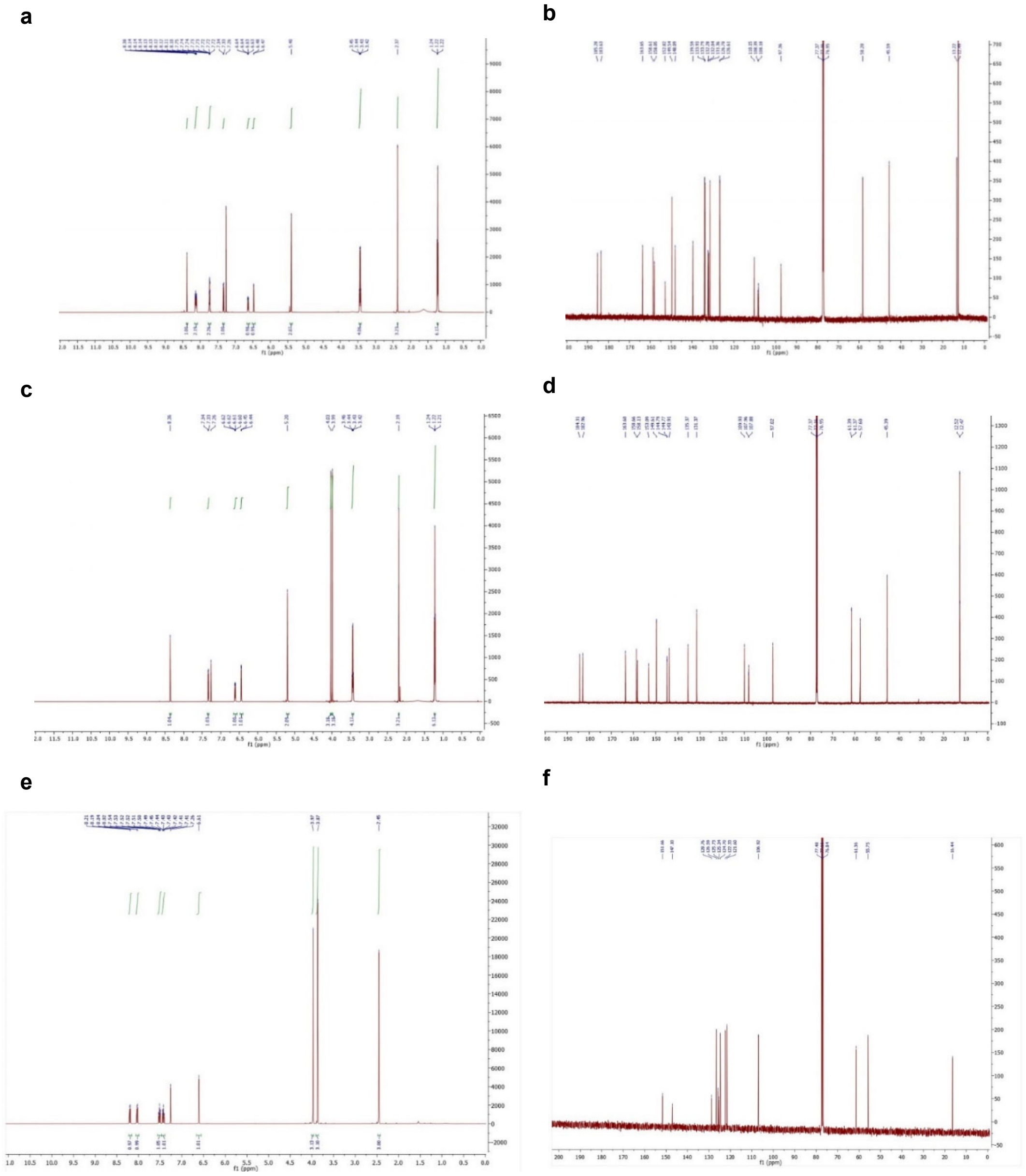

Supplementary Fig 2. NMR spectrums of dimethylmenadione, VK-coumarin and CoQ-coumarin.

a. ^{1}H NMR spectrum of VK-coumarin in $CDCl_3$.

b. ^{13}C NMR spectrum of VK-coumarin in $CDCl_3$.

c. ^{1}H NMR spectrum of CoQ-coumarin in $CDCl_3$.

d. ^{13}C NMR spectrum of CoQ-coumarin in $CDCl_3$.

e. ^{1}H NMR spectrum of 1,4-dimethoxy-2-methylnaphthalene in $CDCl_3$.

f. ^{13}C NMR spectrum of 1,4-dimethoxy-2-methylnaphthalene in $CDCl_3$.

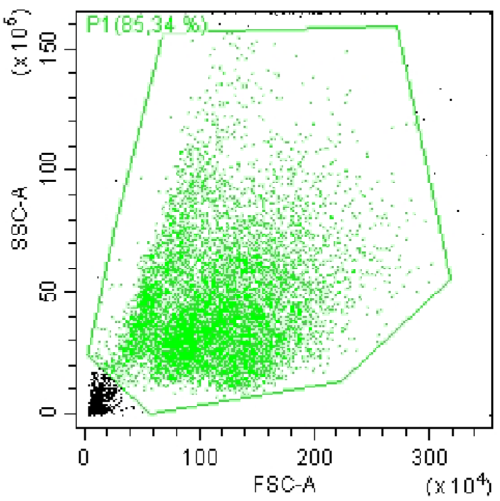

Supplementary Fig 3. An example of the gating strategy in Bodipy C-11 assay
