# Supplementary material for: A non-canonical vitamin K cycle is a potent ferroptosis suppressor
Source: Nature. 2022 Aug 3;608(7924):778–83. doi: 10.1038/s41586-022-05022-3 (PMC9402432; doi:10.1038/s41586-022-05022-3)
Supplement: Supplementary file 2 — Reporting Summary [file 41586_2022_5022_MOESM2_ESM.pdf]

nature portfolio

Corresponding author(s): Marcus Conrad

Last updated by author(s): May 21, 2022

Reporting Summary

Nature Portfolio wishes to improve the reproducibility of the work that we publish. This form provides structure for consistency and transparency in reporting. For further information on Nature Portfolio policies, see our Editorial Policies and the Editorial Policy Checklist.

Statistics

For all statistical analyses, confirm that the following items are present in the figure legend, table legend, main text, or Methods section.

n/a	Confirmed	
☐	☒	The exact sample size (*n*) for each experimental group/condition, given as a discrete number and unit of measurement
☐	☒	A statement on whether measurements were taken from distinct samples or whether the same sample was measured repeatedly
☐	☒	The statistical test(s) used AND whether they are one- or two-sided<br>*Only common tests should be described solely by name; describe more complex techniques in the Methods section.*
☒	☐	A description of all covariates tested
☐	☒	A description of any assumptions or corrections, such as tests of normality and adjustment for multiple comparisons
☐	☒	A full description of the statistical parameters including central tendency (e.g. means) or other basic estimates (e.g. regression coefficient) AND variation (e.g. standard deviation) or associated estimates of uncertainty (e.g. confidence intervals)
☐	☒	For null hypothesis testing, the test statistic (e.g. *F*, *t*, *r*) with confidence intervals, effect sizes, degrees of freedom and *P* value noted<br>*Give P values as exact values whenever suitable.*
☒	☐	For Bayesian analysis, information on the choice of priors and Markov chain Monte Carlo settings
☒	☐	For hierarchical and complex designs, identification of the appropriate level for tests and full reporting of outcomes
☒	☐	Estimates of effect sizes (e.g. Cohen's *d*, Pearson's *r*), indicating how they were calculated

Our web collection on statistics for biologists contains articles on many of the points above.

Software and code

Policy information about availability of computer code

Data collection	CytExpert v2.4 (Beckman Coulter,), Image Lab v6.0 (Biorad), SoftMax Pro v7 (Molecular Devices), Eve v1.8.2 (Nanolive)
Data analysis	GraphPad Prism v9 (GraphPad Software), JMP v15 (SAS Institute Inc.), Flow Jo v10 software (Treestar, Inc), Skyline v21.1 (PMID 31984744), MetaboAnalyst online platform v5.0 (PMID 31756036), Genesis v1.8.1 (Bioinformatics TU-Graz), LipidLynxX system 0.9.24 (doi:10.1101/2020.04.09.033894), Image J v1.53 (NIH), ZEISS Axio Vision software AxioVs v4.9 (Carl Zeiss), ForeCyt software v8 (Sartorius).

For manuscripts utilizing custom algorithms or software that are central to the research but not yet described in published literature, software must be made available to editors and reviewers. We strongly encourage code deposition in a community repository (e.g. GitHub). See the Nature Portfolio guidelines for submitting code & software for further information.

Data

Policy information about availability of data

All manuscripts must include a data availability statement. This statement should provide the following information, where applicable:

- Accession codes, unique identifiers, or web links for publicly available datasets
- A description of any restrictions on data availability
- For clinical datasets or third party data, please ensure that the statement adheres to our policy

All data are available within the Article and the Supplementary Information, and from the corresponding author on reasonable request. Gel source images are shown in Supplementary Fig. 1. All source data are provided with this paper. Epilipidomics data are available at MASSIVE (https://massive.ucsd.edu/) under accession number MSV000089489.

Field-specific reporting

Please select the one below that is the best fit for your research. If you are not sure, read the appropriate sections before making your selection.

☒ Life sciences ☐ Behavioural & social sciences ☐ Ecological, evolutionary & environmental sciences

For a reference copy of the document with all sections, see nature.com/documents/nr-reporting-summary-flat.pdf

Life sciences study design

All studies must disclose on these points even when the disclosure is negative.

Sample size	For in vitro experiments, sample sizes were determined based on previous similar studies that have given statistically significant results (PMID: 31634899). The number of animals studied per treatment group was determined based on our preliminary data and previous similar studies that have given statistically significant results (PMID: 34285231 and 25402683), and respects the limited use of animal models in line with the 3R recommendations: Replacement, Reduction, Refinement.
Data exclusions	No data exclusions.
Replication	The experimental findings were reproduced as validated by at least three independent experiment in Fig1a-d and f, Fig2a-f, Fig 4a, Extended Fig1b, Extended Fig 5a-c, 6a-e, 7b-h, 9e and 10b; and at least two independent experiments in Fig 1g-i, Fig 3a-d, Fig4c, Extended Fig 1a-h, Extended Fig 8b-f, Fig 9b-g.
Randomization	For animal studies, mice were randomized into separate cages. Sex-matched littermates were used and experiments were intended to test a single variable. For in vitro studies, samples were randomized, when possible, prior running.
Blinding	For animal study, mice were given a number prior to data collection and analysis. Data was collected and analyzed blindly. For in vitro experiments, investigators were not blinded, as standard in this manner of study, which contained multiple steps requiring distinct operations for accuracy and precision precluding blinding to experimental variables.

Reporting for specific materials, systems and methods

We require information from authors about some types of materials, experimental systems and methods used in many studies. Here, indicate whether each material, system or method listed is relevant to your study. If you are not sure if a list item applies to your research, read the appropriate section before selecting a response.

Materials & experimental systems

n/a	Involved in the study	
☐	☒	Antibodies
☐	☒	Eukaryotic cell lines
☒	☐	Palaeontology and archaeology
☐	☒	Animals and other organisms
☒	☐	Human research participants
☒	☐	Clinical data
☒	☐	Dual use research of concern

Methods

n/a	Involved in the study	
☒	☐	ChIP-seq
☐	☒	Flow cytometry
☒	☐	MRI-based neuroimaging

Antibodies

Antibodies used	GPX4 (1:1000 for WB, 1:100 for IHC, ab125066, Abcam), 4-HNE (1 µg/mL for WB and 0.5 µg/mL for IHC, MHN-20P, JaICA), human FSP1 (1:1000, sc-377120, Santa Cruz Biotechnology), mouse FSP1 (1:100, clone AIFM2 1A1 rat IgG2a, and 1:1 clone AIFM2 14D7 IgG2b supernatant of hybridoma, developed in-house), VKORC1 (1:1000, ab206656, Abcam), GGCX (1:1000, ab197982, Abcam), β-actin-HRP (1:5000, A3854, Sigma-Aldrich), valosin containing protein (VCP, 1:10000, ab11433, Abcam), anti-KIM-1 (1:200, AF1817, R&D), anti-cleaved caspase-3 (1:100, 9661, Cell Signaling), anti-Gr1-FITC antibody (0.5mg/mL, 553127, BD Pharmingen), goat anti-Rat Alexa Fluor 488 IgG (H+L) (1:500, A-11006, Invitrogen), biotinylated goat anti-rabbit IgG (1:250; BA-1000, Vector Laboratories), biotinylated goat anti-mouse-IgG (1:200; BA-9200, Vector Laboratories), biotinylated donkey anti-goat-IgG diluted (1:500; 208000, Abcam), Histofine Simple Stain MAX PO (R) Anti-Rabbit (414141F, Nichirei), and anti-His antibody clone 3D5 (prepared in-house as described in a previous publication; PMID 8994661).
Validation	GPX4 antibody (ab125066, Abcam) and VCP (Abcam) were validated for WB using mouse and human cells samples in a previous publication (PMID: 31634899).<br>Human FSP1 (sc-377120) was validated for WB using human cells samples in a previous publication (PMID: 31634899).<br>4-HNE (MHN-20P) was validated for IHC and WB using mouse samples on the manufacturer's website (https://www.jaica.com/e/products_lipid_4hne_ab.html).<br>VKORC1 (ab206656) for WB using human cell samples was validated on the manufacturer's website (https://www.abcam.com/vkorc1-antibody-epr20245-ab206656.html).

GGCX (ab197982) for WB using human cell samples was validated on the manufacturer's website (https://www.abcam.com/ggcx-antibody-ab197982.html).
β-actin-HRP (A3854) was validated for WB using mouse and human cell samples on the manufacturer's website (https://www.sigmaaldrich.com/DE/en/product/sigma/a3854).
KIM-1 (AF1817) for IHC using mouse samples was validated in a previous publication (PMID: 31767624).
Cleaved caspase-3 (9661) for IHC using mouse samples was validated in a previous publication (PMID: 30672316).
Gr1 antibody (553127) for IHC using mouse samples was validated in a previous publication (PMID: 31718093).
Anti-His antibody (clone 3D5) for capture of his-tagged proteins was validated in a previous publication (PMID: 8994661)
FSP1 antibody (clone AIFM2 1A1 rat IgG2a, and clone AFM2 14D7 IgG2b, developed in-house) has been validated for WB in this study in Extended Data Fig 9a (clone 1A1) and Extended Data Fig 10b (clone 14D7).

Eukaryotic cell lines

Policy information about cell lines

Cell line source(s)	4-OH-TAM-inducible Gpx4-/- murine immortalized fibroblasts (Pfa1) were reported previously (PMID: 18762024). HT-1080 (CCL-121), 786-O (CRL-1932), A375 (CRL-1619), B16F10 (CRL-6475), H9C2 (CRL-1446), NRK49F (CRL-1570), C2C12 (CRL-1772), HepG2 (HB-8065), Jurkat (TIB-152), L929 (CCL-1), HEK293T (CRL-3216) and P3X63-Ag8.653 (CRL-1580) cells were obtained from ATCC. Panc-1 cells were obtained from Cell Resource Center for Biomedical Research, Institute of Development, Aging and Cancer, Tohoku University (Sendai, Japan). THP-1 cells were obtained from DSMZ (Germany). HT-22 cells were purchased from Millipore (SCC129).
Authentication	None of the cell lines used were authenticated.
Mycoplasma contamination	All cell lines were tested negative for mycoplasma contamination.
Commonly misidentified lines (See ICLAC register)	No commonly misidentified cell lines were used.

Animals and other organisms

Policy information about studies involving animals; ARRIVE guidelines recommended for reporting animal research

Laboratory animals	C57BL/6J male mice (8 to 10-week old) were obtained from Charles River (Sulzfeld, Germany).<br>C57BL/6N male mice (8 to 12-week old) were obtained from Charles River (Sulzfeld, Germany) and CLEA Japan (Tokyo, Japan).<br>Alb-CreERT2 male mice were provided by Prof. Pierre Chambon (Illkirch, France).<br>Gpx4fl/fl mice were reported previously (PMID: 25402683).<br>Alb-CreERT2;Gpx4fl/+ mice were generated by crossing Gpx4fl/fl mice and Alb-CreERT2 mice in the animal facility in our institute.<br>Alb-CreERT2;Gpx4fl/fl (male and female, 8 to 10-week old) were used in the analysis.<br>Fsp1-/- mice (i.e., B6.129-Aifm2tm1Marc/Ieg) were obtained from INFRAFRONTIER (https://www.infrafrontier.eu; EM:05283).<br>Fsp1+/+, +/- and -/- mice (male and female, 8 to 16-week old) were used in the analysis.<br>Gpx4 fl/fl and Fsp1-/- mice were on a congenic C57BL/6J background.<br>Wister rats (RjHan:Wi, female, age 160 days) were obtained from Javier Labs (France).<br>Mice were kept under standard conditions with water and food ad libitum and in a controlled environment (22 ± 2°C, 55 ± 5% humidity, 12 h light/dark cycle).
Wild animals	The study did not involve wild animals.
Field-collected samples	The study did not involve field-collected samples.
Ethics oversight	All experiments were performed in compliance with the German Animal Welfare Law and have been approved by the institutional committee on animal experimentation and the government of Upper Bavaria (approved No. ROB-55.2-2532-Vet_02-18-13 and ROB-55.2-2532.Vet_03-17-68) and the State of Bavaria (permission granted by the government of Lower Franconia, approved No. 54-2532.1-19/13), the Landesdirektion Sachsen (TVV07/2021) involving an independent ethics committee, and the Animal Committee of Tohoku University (approved No. No. 2019-BeA012, 2019-BeA014 and 2019PhA-010-01).

Note that full information on the approval of the study protocol must also be provided in the manuscript.

Flow Cytometry

Plots

Confirm that:

☒ The axis labels state the marker and fluorochrome used (e.g. CD4-FITC).

☒ The axis scales are clearly visible. Include numbers along axes only for bottom left plot of group (a 'group' is an analysis of identical markers).

☒ All plots are contour plots with outliers or pseudocolor plots.

☒ A numerical value for number of cells or percentage (with statistics) is provided.

Methodology

Sample preparation	Pfa1 cells (50,000 cells/well) were seeded on 6-well dishes one day prior to the experiment. On the next day, cells were treated with 0.3 µM RSL3 to induce ferroptosis. Three hours later, cells were incubated with 1.5 µM of BODIPY 581/591 C11 (ThermoFisher) for 30 min at 37 °C. Subsequently, cells were trypsinized, resuspended in 300 µL of Hanks' balanced salt solution (HBSS, Gibco), strained through a 40 µm cell strainer (Falcon tube with cell strainer CAP), and then analyzed using a flow cytometer (CytoFLEX, Beckman Coulter) with a 488-nm laser paired with a 530/30 nm bandpass filter.
Instrument	CytoFLEX (Beckman Coulter)
Software	CytExpert v2.4 was used for data collection. FlowJo v10 was used for data analysis.
Cell population abundance	At least 8,000 cells were analyzed for each sample.
Gating strategy	Cell populations were separated from cellular debris using FSC and SSC.

☒ Tick this box to confirm that a figure exemplifying the gating strategy is provided in the Supplementary Information.
